# Splice Variant of *Spalax* Heparanase Skipping Exon 12

**DOI:** 10.3390/genes15081039

**Published:** 2024-08-07

**Authors:** Nicola J. Nasser, Eviatar Nevo, Aaron Avivi

**Affiliations:** 1 Department of Radiation Oncology, NYC Health + Hospitals/Elmhurst, Queens, NY 11373, USA; 2 The Mount Sinai Hospital, Icahn School of Medicine at Mount Sinai, New York, NY 10029, USA; 3 Institute of Evolution, University of Haifa, Abba Khoushy Ave. 199, Haifa 3498838, Israel; nevo@research.haifa.ac.il

**Keywords:** *Spalax*, alternative splicing, heparanase, hypoxia, angiogenesis

## Abstract

The subterranean blind mole rat, *Spalax*, has evolved significantly over 47 million years to thrive in its underground habitat. A key enzyme in this adaptation is heparanase, which degrades heparan sulfate (HS) in the extracellular matrix (ECM), facilitating angiogenesis and releasing growth factors for endothelial cells. *Spalax* heparanase has various splice variants influencing tumor growth and metastasis differently. We report a novel splice variant from a hypoxia-exposed kidney sample resulting from exon 12 skipping. This variant maintains the translation frame but lacks enzymatic activity, offering insights into *Spalax*’s unique adaptations.

## 1. Introduction

The subterranean blind mole rat (*Spalax*) is a fascinating mammal that spends the majority of its life living underground. Over 47 million years, *Spalax* has evolved unique adaptations to its subterranean environment [1]. One notable adaptation is the atrophy of its eyes, as living in darkness in subterranean tunnels made sight less essential [2]. Despite this, *Spalax* is still able to distinguish between light and darkness [3]. *Spalax*’s limbs have also become shorter to accommodate the narrow tunnels [2]. However, *Spalax*’s muscles are much more developed and have a higher density of blood vessels compared with other rodents [4]. In addition, *Spalax* has developed several extraordinary abilities to navigate and communicate underground. It can perceive and utilize the Earth’s magnetic field to orient itself in space while digging underground [5,6]. Furthermore, *Spalax* has developed seismic communication using low-frequency and patterned substrate-borne vibrations generated by head thumping [7]. *Spalax* lives much longer than rats and mice, reaching up to 20 years. This remarkable longevity is potentially supported by the damage-free shortening of telomeres [8]. These unique adaptations have allowed *Spalax* to thrive in its subterranean habitat and provide valuable insights into how species adapt to extreme environments.

In Israel, the genus *Spalax* is part of the superspecies *Spalax ehrenbergi* [9]. Four distinct Israeli species share similar morphology but differ in their diploid chromosome numbers. Specifically, *Spalax galili*, *S. golani*, *S. carmeli*, and *S. judaei* have 52, 54, 58, and 60 chromosomes, respectively [9]. One unique characteristic of *Spalax* is its ability to maintain high levels of metabolic activity in both hypoxic and hypercapnic conditions [10]. Moreover, *Spalax* has a lower coronary artery resistance in its heart compared with other rodents [11]. In terms of its lipid profile, *Spalax* has high levels of high-density lipoproteins (HDLs) and low levels of low-density lipoproteins (LDLs) and triglycerides as compared to humans [12]. Additionally, *Spalax* has higher antioxidant serum content than both humans and mice [12].

Heparan sulfates (HSs) are polysaccharides attached to core proteins found in the extracellular matrix (ECM) and on the cell surface [13]. HS of *Spalax* tissues exhibit higher sulfation levels compared with murine tissues [13]. Heparanase is an enzyme that cleaves HS side chains from heparan sulfate proteoglycans [14]. Heparanase is normally expressed in human platelets, immune cells, and placenta [15,16,17]. Most malignant tumors express heparanase [18,19,20], including prostate [21,22], lung [23], breast [24], colon [25], and kidney [26] cancers.

Heparanase 2 (HPSE2) was initially mislabeled as heparanase due to its structural similarity to heparanase (also known as heparanase I, HPSE1), as alignment of the coding regions revealed 35% overall identity, underscoring some structural similarity [27]. However, HPSE2 lacks the enzymatic activity to cleave heparan sulfate [28,29,30]. Mutations in HPSE2 are linked to urofacial syndrome, a rare genetic disorder affecting the urinary and facial systems [31,32].

In our previous study, we reported on the successful cloning of *Spalax* heparanase and its ability to degrade heparan sulfate [33]. We also identified multiple splice variants of heparanase in *Spalax*. For example, splice 7 results from the skipping of exon 7, while splice 36 is generated by skipping parts of exons 3 and 6 and all of exons 4 and 5. Splice 36 functions as a dominant negative to wild-type heparanase, thereby suppressing HS degradation, tumor growth, and metastasis [34]. In addition, splice 67 arises from the skipping of exons 6 and 7, and splice 612 from skipping exons 6 to 12. These two splice variants generate truncated heparanase proteins that share the same N-terminus as the native wild-type enzyme, but each possesses a unique C-terminus [35]. In the current study, we describe a new splice variant, splice 12 of *Spalax* heparanase, generated by the skipping of exon 12 and is devoid of heparanase enzymatic activity.

## 2. Materials and Methods

### 2.1. Animals

The *Spalax* used in this study were captured from their natural habitat by removing the dirt covering their tunnel openings. Once the animals were detected near the entrance trying to re-block it with dirt, the hunters blocked their way back and captured them. Since *Spalax* do not reproduce in captivity, maintaining wild-caught animals is essential for research. The captured *Spalax* were then housed individually at the animal facility of the Institute of Evolution, University of Haifa, Israel. They were maintained under controlled conditions at 22–24 °C and fed a diet of carrots and apples. The study utilized adult *Spalax judaei* from the Anza population, weighing between 100 to 150 g. The animals were exposed to a hypoxic pulse (6% O_2_ for 3 h) and subsequently euthanized by injection of Ketaset CIII (Fort Dodge Animal Health, Fort Dodge, IA, USA) at a dose of 5 mg/kg body weight. Whole organs were removed and immediately frozen in liquid nitrogen.

### 2.2. RNA and cDNA Preparation

Total RNA was meticulously extracted from tissues using TRI Reagent (Molecular Research Center, Cincinnati, OH, USA) according to the manufacturer’s detailed instructions. Subsequently, cDNA was synthesized by reverse transcription of 1 µg of total RNA with Moloney murine leukemia virus reverse transcriptase (Promega, Madison, WI, USA), utilizing oligo(dT)15 and random primers to ensure comprehensive coverage of mRNA transcripts [33,36].

### 2.3. Cloning of Spalax Heparanase Splice Variants

To clone *Spalax* heparanase splice variants, cDNAs were prepared from kidney tissues, and *Spalax*-specific primers were used [33]. To clone splice 12, forward primer Mf: GGTCAACCTCGAGGAAAGACAGTTAA and reverse primer s3′Lb: TCATAGACAAGCAGCAACTTTGGCATTTC (Sigma Genosys, Rehovot, Israel) were utilized. PCR was performed using TaqDNA polymerase (Qbiogene, Illkirch, France) and kidney cDNA as a template. The presence of splice 12 was confirmed by PCR utilizing sHep1742f and s3′Lb primers. The amplified band was sub-cloned into the pGEM-Teasy vector, sequenced with gene- and vector-specific primers using an automated DNA sequencer (ABI Prism model 310 Genetic Analyzer; Perkin Elmer, Foster City, CA, USA), and then constructed into the pcDNA3 expression vector (Invitrogen, Leek, The Netherlands) containing the full-length *Spalax* heparanase by means of digestion and ligation with site-specific restriction enzymes and T4 ligase (Promega, Madison, WI, USA).

### 2.4. Cells and Transfections

Uppsala 87 Malignant Glioma (U87) cells were cultured in Dulbecco’s Modified Eagle’s Medium (DMEM) containing 4.5 g/L glucose, 10% fetal calf serum (FCS), and antibiotics, as described [34]. Cells were grown in 60 mm tissue culture dishes and transfected with a total of 1–2 µg of plasmid DNA mixed with 6 µL of Fu Gene transfection reagent (Roche Applied Science, Mannheim, Germany) and 94 µL of DMEM. Transiently transfected cells were obtained after 24–48 h of incubation at 37 °C. Stable populations of transfected cells were selected with G418 (Sigma Genosys, Rehovot, Israel).

We chose U87 cells for transfections and heparanase enzymatic activity assays because these cells do not have endogenous expression of heparanase. This lack of endogenous heparanase ensures that any observed enzymatic activity or effects on cell behavior can be attributed solely to the transfected heparanase constructs, thereby providing a clear and uncontaminated assessment of the splice variants’ functional properties. This makes U87 cells an ideal model system for studying the specific activities of heparanase splice variants.

### 2.5. Western Blot Analysis

Cells (2 × 10^6^) transfected with either *Spalax* wild-type, splice 7, splice 12, splice 36 heparanase, or insert-free pcDNA3 vector alone were lysed in 1 mL lysis buffer containing 50 mM Tris-HCl, pH 7.4, 150 mM NaCl, 0.5% Triton X-100, and a mixture of protease inhibitors (Roche Applied Science, Mannheim, Germany). Heparanase was concentrated by incubating the cell lysate (4 °C, 1 h) with ConA beads (Amersham Biosciences, Uppsala, Sweden) and washing (×2) with PBS. The beads were boiled (3 min) in sample buffer and centrifuged, and the supernatant was subjected to SDS-PAGE and immunoblot analysis using polyclonal anti-heparanase antibodies #1453 (1:2500), as described [33,37]. The polyclonal anti-heparanase antibody #1453 was generated against the entire 65 kDa heparanase precursor isolated from the conditioned medium of heparanase-transfected 293 cells. This antibody recognizes both the latent and active forms of heparanase [38]. Immunoreactive bands were detected by the enhanced chemiluminescence reagent, as described [17].

### 2.6. Heparanase Activity

Cell lysates prepared from 1 × 10^6^ cells by three cycles of freezing and thawing in heparanase reaction buffer (20 mM phosphate–citrate buffer, pH 6.0/1 mM DTT/1 mM CaCl2/50 mM NaCl) were incubated (4 h, 37 °C, pH 6.0) with ^35^S-labeled ECM, prepared as described [17]. The incubation medium containing ^35^S-labeled HS degradation fragments was analyzed by gel filtration on a Sepharose CL-6B column. Fractions (0.2 mL) were eluted with PBS, and their radioactivity was counted in a β-scintillation counter. Degradation fragments of HS side chains were eluted from Sepharose 6B at 0.5 < Kav < 0.8 (peak II, fractions 20–30) [17].

## 3. Results

### 3.1. Cloning of Splice Variant #12 of Spalax Heparanase

**Figure 1:**
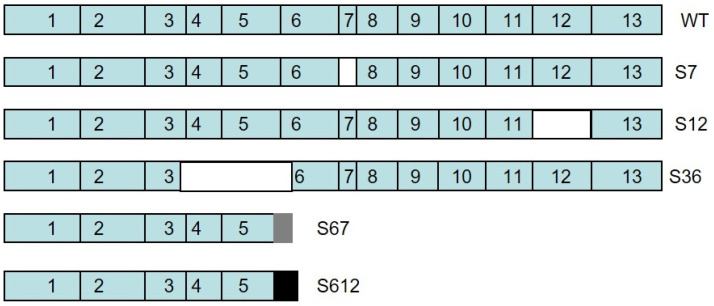
Schematic drawing of *Spalax* wild-type (WT) heparanase and its splice variants. Splice 7 (S7), splice 12 (S12) and splice 36 (S36), results of skipping of nucleotides numbers that are a multiple of three, and maintain the reading frame. Splice 67 (S67) and splice 612 (S612) skips nucleotide numbers that are not a multiple of three, resulting in a frameshift, and proteins that has unique C-terminals sequences.

**Figure 2:**
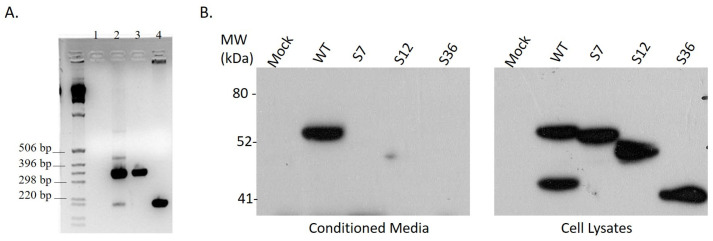
(**A**) Cloning of splice variant #12 of *Spalax* heparanase. Semi-quantitative RT-PCR using *Spalax*-specific primers located around the heparanase cDNA region encoded by exon 12. Bands of 336 bp represent the wild-type enzyme, while those of 189 bp represent its splice 12 form. Lane 1: reaction mixture alone; Lane 2: cDNA of kidney; Lanes 3 and 4, plasmids containing the cDNA sequence of the wild-type *Spalax* heparanase and the splice 12 variant, respectively. (**B**) Western blot analysis. U87 cells transfected with Mock empty plasmid, or plasmids containing wild-type (WT), splice 7 (S7), splice 12 (S12), or splice 36 (S36) of *Spalax* heparanase were cultured, as described in Methods. Western blot analysis of conditioned media (**left**) and cell lysates (**right**) was performed using polyclonal anti-heparanase antibodies #1453.

Splice variant #12 of *Spalax* heparanase was cloned from a kidney of *Spalax judaei* of the Anza population that was exposed to a 3 h pulse of hypoxia with 6% O_2_. This variant is different from the wild-type heparanase and other splice variants, as it results from splicing out of exon 12 [39] (nucleotides 1770–1916) (Figure 1). As a result, the cDNA of splice variant #12 is 147 base pairs shorter than the wild-type sequence, but it does not cause any frameshift. PCR products were amplified using kidney cDNA as a template and primers designed around the exon 12 segment. Gel electrophoresis of these products revealed both the wild-type and spliced forms (Figure 2A, lane 2).

### 3.2. Expression of Spalax Heparanase Splice Variants in U87 Cells

**Figure 3:**
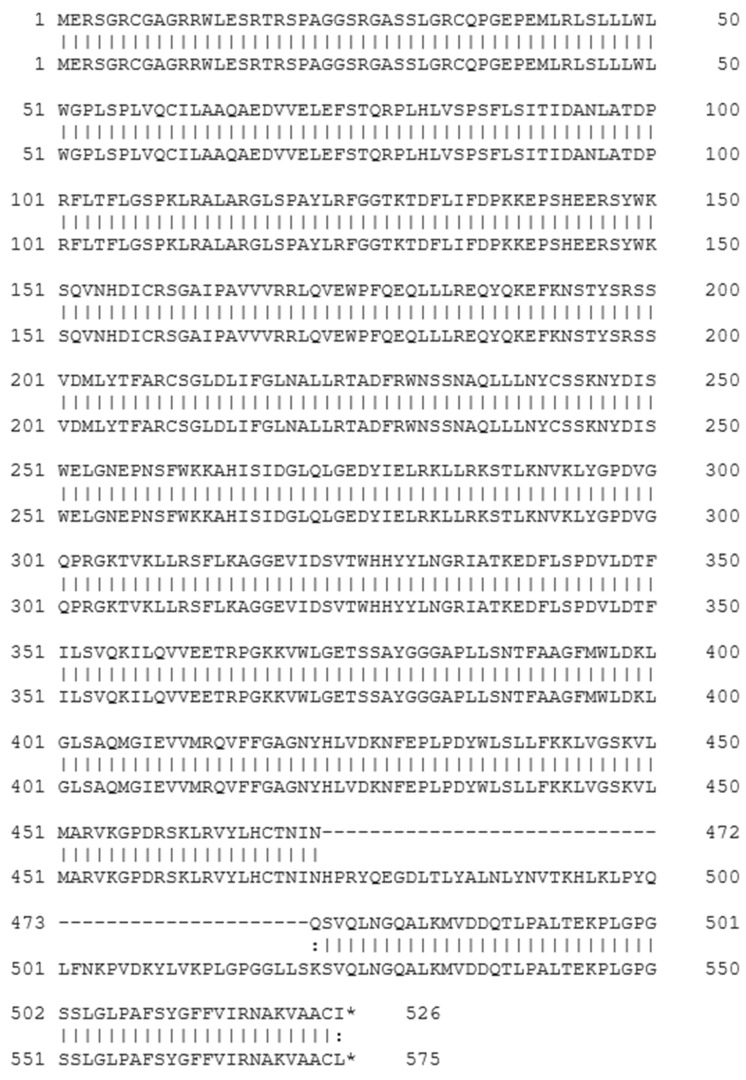
Amino acid sequences of splice 12 (top) and wild-type (bottom) *Spalax judaei* heparanase. Splice 12 lacks 49 amino acids compared with the wild-type heparanase enzyme—represent amino acids missing in splice 12: represents conserved substitutions (amino acids with similar properties), and * represent a stop codon.

Splice variant 12 of *Spalax* heparanase had a shorter amino acid sequence, lacking 49 amino acids compared with the wild-type protein. The splicing out of exon 12 results in a substitution of Lys473 with Gln at the junction of exons 11 and 13 (Figure 3). To examine the expression and secretion of different heparanase splice variants, U87 cells were transfected with plasmids containing the Mock empty vector, wild-type (WT), splice 7 (S7), splice 12 (S12), or splice 36 (S36) of *Spalax* heparanase. Western blot analysis was performed using polyclonal anti-heparanase antibodies #1453 to detect heparanase in the conditioned media and cell lysates (Figure 2B). The results showed that only *Spalax* WT heparanase was detected in the conditioned media in its latent form, while the cell lysates contained both the latent and active forms of the protein. The splice variants S7, S12, and S36 were expressed as a single band in the cell lysates, whereas the wild-type *Spalax* heparanase was expressed as two bands, representing its latent and active forms. (Figure 2B).

### 3.3. Heparanase Enzymatic Activity

**Figure 4:**
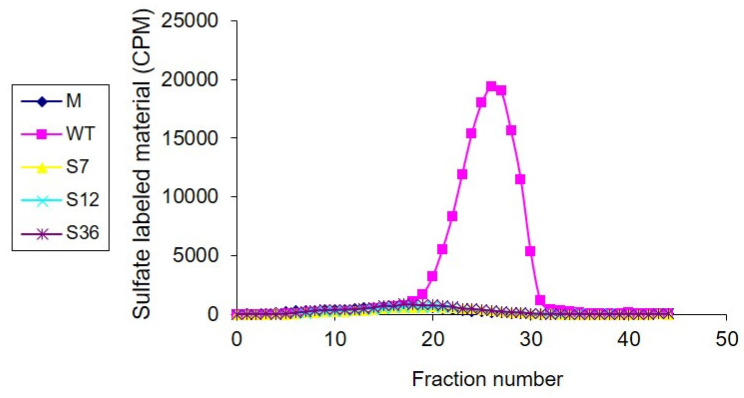
U87 cells transfected with Mock empty plasmid (M), or plasmids containing wild-type (WT), splice 7 (S7), splice 12 (S12), or splice 36 (S36) of *Spalax* heparanase were lysed and incubated (4 h, 37 °C, pH 6.0) with intact, naturally produced, sulfate-labeled ECM. Labeled degradation fragments released into the incubation medium were then analyzed by gel filtration on Sepharose 6B.

U87 cells were transfected with either an empty plasmid (M), plasmids containing wild-type (WT) *Spalax* heparanase, splice variants 7 (S7), 12 (S12), or 36 (S36). Cell lysates were incubated with naturally produced, sulfate-labeled extracellular matrix (ECM) for 4 h at 37 °C (pH 6.0). Degradation fragments released into the medium were then analyzed by gel filtration on Sepharose 6B. Results showed that only the lysates of cells transfected with WT *Spalax* heparanase were able to release low-molecular-weight-labeled degradation fragments of HS from the ECM. In contrast, lysates of cells transfected with S7, S12, or S36 *Spalax* heparanase or Mock empty plasmid (M) did not result in degradation of HS (Figure 4). 

## 4. Discussion

Heparanase is an enzyme that specifically degrades heparan sulfate [16,36]. It is highly expressed in platelets, and upon activation at sites of injury, the secreted enzyme degrades the endogenous heparin/HS found in the blood, thereby promoting blood clotting [16]. In addition to its roles in platelet function and wound healing, heparanase has been implicated in various pathological processes, including inflammation, angiogenesis, metastasis, and tumor growth [16,17]. Heparanase expression has been reported in various cell types, including leukocytes, endothelial cells, and cancer cells [17,21,23,24,25,37]. In cancer, heparanase promotes tumor growth and metastasis by facilitating tumor cell invasion and angiogenesis through degradation of the extracellular matrix and the release of pro-angiogenic and growth-promoting factors [15,17]. Heparanase inhibitors have been developed as potential anti-cancer agents, and clinical trials are currently underway to evaluate their efficacy [40,41]. Overall, heparanase is a multifaceted enzyme that plays important roles in various physiological and pathological processes, and its regulation and therapeutic targeting have significant implications for human health.

We previously reported the expression of wild-type heparanase in *Spalax* kidney, highlighting the critical role of this organ in maintaining homeostasis and adapting to hypoxic conditions [33]. The kidneys’ functions in blood filtration and oxygen regulation make them an ideal model for studying physiological responses to hypoxia [42,43]. During our investigations of heparanase activity in various kidney tissues of *Spalax*, we identified a novel splice variant, S12. This discovery underscores the unique genetic adaptations of *Spalax* to its subterranean habitat and emphasizes the significance of heparanase splice variants in these processes.

Transfecting U87 cells with *Spalax* heparanase splice variants S7, S12, or S36 results in specific functional changes: S7 increases tumor growth despite lacking enzymatic activity [34]; S12, reported for the first time in this article, lacks enzymatic activity, and its effect on cell behavior is not fully characterized yet; and S36 reduces tumor growth by inhibiting heparan sulfate degradation [34]. This highlights the diverse functional roles of heparanase splice variants and their potential implications in tumor biology [15].

The cloning of splice variant 12 of *Spalax* heparanase provides an opportunity for further research into the function and role of heparanase in health and disease. Studying the splice variants of heparanase can enhance our understanding of the protein’s diversity and its potential roles in various cellular processes. Furthermore, the ability to express and study different splice variants of heparanase in vitro can provide insights into their differential activities, which may have implications for developing targeted therapeutics for cancer, inflammation, kidney dysfunction, viral infections, and other pathologies.

*Spalax* underwent genetic adaptations to survive the harsh underground environment. One notable adaptation is the evolution of a unique form of hemoglobin, which has a higher affinity for oxygen than the hemoglobin found in other mammals. This adaptation allows *Spalax* to extract oxygen more efficiently from the low-oxygen environment in its burrow [4,44,45]. Additionally, *Spalax* has adaptations in its metabolism, such as a lower metabolic rate, which allow it to conserve energy and survive periods of food scarcity [2,44,46]. *Spalax* also has an enhanced immune system, likely due to the high pathogenicity in its underground environment [1,9,47]. These adaptations in *Spalax* have led to interest in studying the molecular mechanisms underlying its unique physiology and potential applications for human health.

Alternative splicing can result in the production of multiple mRNA variants of the same gene, leading to different protein isoforms with varying functions or properties [48,49]. Exon skipping, partial skipping of an exon, or intron retention can all contribute to alternative splicing, and the resulting protein isoforms likely have a different amino acid sequence and structure as compared with the original protein [34,49]. When the skipped nucleotide number is not a multiple of three, a frameshift occurs, and the resulting protein has a unique C-terminal sequence [35]. This can affect the protein’s enzymatic activity, cellular localization, or interactions with other molecules [15,34,35,36].

Splice #12 variant results from the splicing out of exon 12, which causes a deletion of 147 base pairs in the cDNA sequence. This deletion maintains the reading frame and results in a protein that is shorter by 49 amino acids compared with the wild-type heparanase. This deletion affects the C-terminus of the protein, which contains functional domains important for heparanase secretion, structural integrity, enzymatic and non-enzymatic activities, interaction with other proteins, and pro-tumorigenic properties [50,51,52,53,54]. 

The discovery of splice variant 12 of *Spalax* heparanase provides an opportunity to further explore the diversity and potential roles of heparanase in various cellular processes, including the adaptation of *Spalax* to its harsh underground environment. Further research into heparanase and its splice variants can provide valuable insights into its regulation and therapeutic targeting for various diseases [55].

## 5. Patents

US patent number: US8093031. US Provisional application number: US60/718354. Patent assigned to Carmel Haifa University Economic Corp Ltd. (Haifa, Israel).

## Data Availability

All sequences cloned were published under the US patent US8093031, available through Google Patents: https://patentimages.storage.googleapis.com/7f/a6/90/922bf795478f24/US8093031.pdf (accessed 6 August 2024).

## References

[1] Fang X., Nevo E., Han L., Levanon E.Y., Zhao J., Avivi A., Larkin D., Jiang X., Feranchuk S., Zhu Y. (2014). Genome-wide adaptive complexes to underground stresses in blind mole rats Spalax. Nat. Commun..

[2] Nevo E. (1999). Mosaic Evolution of Subterranean Mammals: Regression, Progression, and Global Convergence.

[3] Avivi A., Albrecht U., Oster H., Joel A., Beiles A., Nevo E. (2001). Biological clock in total darkness: The Clock/MOP3 circadian system of the blind subterranean mole rat. Proc. Natl. Acad. Sci. USA.

[4] Avivi A., Shams I., Joel A., Lache O., Levy A.P., Nevo E. (2005). Increased blood vessel density provides the mole rat physiological tolerance to its hypoxic subterranean habitat. FASEB J. Off. Publ. Fed. Am. Soc. Exp. Biol..

[5] Kimchi T., Terkel J. (2001). Magnetic compass orientation in the blind mole rat Spalax ehrenbergi. J. Exp. Biol..

[6] Malewski S., Begall S., Schleich C.E., Antenucci C.D., Burda H. (2018). Do subterranean mammals use the Earth’s magnetic field as a heading indicator to dig straight tunnels?. PeerJ.

[7] Nevo E., Heth G., Pratt H. (1991). Seismic communication in a blind subterranean mammal: A major somatosensory mechanism in adaptive evolution underground. Proc. Natl. Acad. Sci. USA.

[8] Adwan Shekhidem H., Sharvit L., Huffman D.M., Manov I., Atzmon G., Shams I. (2023). Damage-Free Shortening of Telomeres Is a Potential Strategy Supporting Blind Mole-Rat Longevity. Genes.

[9] Nevo E., Ivanitskaia E., Beiles A. (2001). Adaptive Radiation of Blind Subterranean Mole Rats.

[10] Widmer H.R., Hoppeler H., Nevo E., Taylor C.R., Weibel E.R. (1997). Working underground: Respiratory adaptations in the blind mole rat. Proc. Natl. Acad. Sci. USA.

[11] Edoute Y., Arieli R., Nevo E. (1988). Evidence for improved myocardial oxygen delivery and function during hypoxia in the mole rat. J. Comp. Physiol. B.

[12] Nasser N.J., Kaplan M., Nevo E., Aviram M. (2009). Lipid profile and serum characteristics of the blind subterranean mole rat, Spalax. PLoS ONE.

[13] Sandwall E., Bodevin S., Nasser N.J., Nevo E., Avivi A., Vlodavsky I., Li J.P. (2009). Molecular structure of heparan sulfate from Spalax. Implications of heparanase and hypoxia. J. Biol. Chem..

[14] Zhang Y., Xiong M., Chen Z., Seabra G., Liu J., Li C., Cui L. (2024). Design Principle of Heparanase Inhibitors: A Combined In Vitro and In Silico Study. ACS Med. Chem. Lett..

[15] Nasser N.J. (2008). Heparanase involvement in physiology and disease. Cell Mol. Life Sci..

[16] Nasser N.J., Fox J., Agbarya A. (2020). Potential Mechanisms of Cancer-Related Hypercoagulability. Cancers.

[17] Vlodavsky I., Friedmann Y., Elkin M., Aingorn H., Atzmon R., Ishai-Michaeli R., Bitan M., Pappo O., Peretz T., Michal I. (1999). Mammalian heparanase: Gene cloning, expression and function in tumor progression and metastasis. Nat. Med..

[18] Jayatilleke K.M., Hulett M.D. (2020). Heparanase and the hallmarks of cancer. J. Transl. Med..

[19] Koganti R., Suryawanshi R., Shukla D. (2020). Heparanase, cell signaling, and viral infections. Cell. Mol. Life Sci..

[20] Mayfosh A.J., Nguyen T.K., Hulett M.D. (2021). The heparanase regulatory network in health and disease. Int. J. Mol. Sci..

[21] Lerner I., Baraz L., Pikarsky E., Meirovitz A., Edovitsky E., Peretz T., Vlodavsky I., Elkin M. (2008). Function of heparanase in prostate tumorigenesis: Potential for therapy. Clin. Cancer Res. Off. J. Am. Assoc. Cancer Res..

[22] Nasser N.J., Klein J., Agbarya A. (2021). Markers of Toxicity and Response to Radiation Therapy in Patients With Prostate Cancer. Adv. Radiat. Oncol..

[23] Zhang Q., Ming J., Li Y., Zhang S., Li B., Qiu X., Wang E. (2009). Heparanase expression correlates with angiogenesis and lymphangiogenesis in human lung cancer. Chin. J. Lung Cancer.

[24] Vornicova O., Naroditsky I., Boyango I., Shachar S.S., Mashiach T., Ilan N., Vlodavsky I., Bar-Sela G. (2018). Prognostic significance of heparanase expression in primary and metastatic breast carcinoma. Oncotarget.

[25] Friedmann Y., Vlodavsky I., Aingorn H., Aviv A., Peretz T., Pecker I., Pappo O. (2000). Expression of heparanase in normal, dysplastic, and neoplastic human colonic mucosa and stroma. Evidence for its role in colonic tumorigenesis. Am. J. Pathol..

[26] Mikami S., Oya M., Shimoda M., Mizuno R., Ishida M., Kosaka T., Mukai M., Nakajima M., Okada Y. (2008). Expression of heparanase in renal cell carcinomas: Implications for tumor invasion and prognosis. Clin. Cancer Res. Off. J. Am. Assoc. Cancer Res..

[27] McKenzie E., Tyson K., Stamps A., Smith P., Turner P., Barry R., Hircock M., Patel S., Barry E., Stubberfield C. (2000). Cloning and expression profiling of Hpa2, a novel mammalian heparanase family member. Biochem. Biophys. Res. Commun..

[28] Gross-Cohen M., Yanku Y., Kessler O., Barash U., Boyango I., Cid-Arregui A., Neufeld G., Ilan N., Vlodavsky I. (2021). Heparanase 2 (Hpa2) attenuates tumor growth by inducing Sox2 expression. Matrix Biol. J. Int. Soc. Matrix Biol..

[29] Vlodavsky I., Hilwi M., Kayal Y., Soboh S., Ilan N. (2024). Impact of heparanase-2 (Hpa2) on cancer and inflammation: Advances and paradigms. FASEB J. Off. Publ. Fed. Am. Soc. Exp. Biol..

[30] Kayal Y., Barash U., Naroditsky I., Ilan N., Vlodavsky I. (2023). Heparanase 2 (Hpa2)- a new player essential for pancreatic acinar cell differentiation. Cell Death Dis..

[31] Lopes F.M., Grenier C., Jarvis B.W., Al Mahdy S., Lène-McKay A., Gurney A.M., Newman W.G., Waddington S.N., Woolf A.S., Roberts N.A. (2024). Human HPSE2 gene transfer ameliorates bladder pathophysiology in a mutant mouse model of urofacial syndrome. eLife.

[32] Daly S.B., Urquhart J.E., Hilton E., McKenzie E.A., Kammerer R.A., Lewis M., Kerr B., Stuart H., Donnai D., Long D.A. (2010). Mutations in HPSE2 cause urofacial syndrome. Am. J. Hum. Genet..

[33] Nasser N.J., Nevo E., Shafat I., Ilan N., Vlodavsky I., Avivi A. (2005). Adaptive evolution of heparanase in hypoxia-tolerant *Spalax*: Gene cloning and identification of a unique splice variant. Proc. Natl. Acad. Sci. USA.

[34] Nasser N.J., Avivi A., Shafat I., Edovitsky E., Zcharia E., Ilan N., Vlodavsky I., Nevo E. (2009). Alternatively spliced Spalax heparanase inhibits extracellular matrix degradation, tumor growth, and metastasis. Proc. Natl. Acad. Sci. USA.

[35] Nasser N.J., Avivi A., Vlodavsky I., Nevo E. (2020). Cloning of two splice variants of Spalax heparanase encoding for truncated proteins. Anti-Cancer Drugs.

[36] Nasser N.J., Avivi A., Shushy M., Vlodavsky I., Nevo E. (2007). Cloning, expression, and characterization of an alternatively spliced variant of human heparanase. Biochem. Biophys. Res. Commun..

[37] Weissmann M., Arvatz G., Horowitz N., Feld S., Naroditsky I., Zhang Y., Ng M., Hammond E., Nevo E., Vlodavsky I. (2016). Heparanase-neutralizing antibodies attenuate lymphoma tumor growth and metastasis. Proc. Natl. Acad. Sci. USA.

[38] Zetser A., Levy-Adam F., Kaplan V., Gingis-Velitski S., Bashenko Y., Schubert S., Flugelman M.Y., Vlodavsky I., Ilan N. (2004). Processing and activation of latent heparanase occurs in lysosomes. J. Cell Sci..

[39] Dong J., Kukula A.K., Toyoshima M., Nakajima M. (2000). Genomic organization and chromosome localization of the newly identified human heparanase gene. Gene.

[40] Cassinelli G., Torri G., Naggi A. (2020). Non-anticoagulant heparins as heparanase inhibitors. Heparanase: From Basic Research to Clinical Applications.

[41] de Boer C., Armstrong Z., Lit V.A., Barash U., Ruijgrok G., Boyango I., Weitzenberg M.M., Schröder S.P., Sarris A.J., Meeuwenoord N.J. (2022). Mechanism-based heparanase inhibitors reduce cancer metastasis in vivo. Proc. Natl. Acad. Sci. USA.

[42] Liu J., Wei Q., Guo C., Dong G., Liu Y., Tang C., Dong Z. (2017). Hypoxia, HIF, and associated signaling networks in chronic kidney disease. Int. J. Mol. Sci..

[43] Kanbay M., Altıntas A., Yavuz F., Copur S., Sanchez-Lozada L.G., Lanaspa M.A., Johnson R.J. (2023). Responses to Hypoxia: How Fructose Metabolism and Hypoxia-Inducible Factor-1a Pathways Converge in Health and Disease. Curr. Nutr. Rep..

[44] Arieli R., Nevo E. (1991). Hypoxic survival differs between two mole rat species (*Spalax ehrenbergi*) of humid and arid habitats. Comp. Biochem. Physiol. A Comp. Physiol..

[45] Sun H., Ye K., Liu D., Pan D., Gu S., Wang Z. (2020). Evolution of hemoglobin genes in a subterranean rodent species (*Lasiopodomys mandarinus*). Biology.

[46] Šumbera R., Lovy M., Nevo E., Okrouhlík J. (2023). Thermal Biology in the Upper Galili Mountain Blind Mole Rat (*Nannospalax galili*) and an Overview of Spalacine Energetics. J. Therm. Biol..

[47] Izraelson M., Metsger M., Davydov A., Shagina I., Dronina M., Obraztsova A., Miskevich D., Mamedov I., Volchkova L., Nakonechnaya T. (2021). Distinct organization of adaptive immunity in the long-lived rodent Spalax galili. Nat. Aging.

[48] Oltean S., Bates D.O. (2014). Hallmarks of alternative splicing in cancer. Oncogene.

[49] Reixachs-Solé M., Eyras E. (2022). Uncovering the impacts of alternative splicing on the proteome with current omics techniques. Wiley Interdiscip. Rev. RNA.

[50] Fux L., Ilan N., Sanderson R.D., Vlodavsky I. (2009). Heparanase: Busy at the cell surface. Trends Biochem. Sci..

[51] Lai N.S., Simizu S., Morisaki D., Muroi M., Osada H. (2008). Requirement of the conserved, hydrophobic C-terminus region for the activation of heparanase. Exp. Cell Res..

[52] Levy-Adam F., Abboud-Jarrous G., Guerrini M., Beccati D., Vlodavsky I., Ilan N. (2005). Identification and characterization of heparin/heparan sulfate binding domains of the endoglycosidase heparanase. J. Biol. Chem..

[53] Levy-Adam F., Miao H.-Q., Heinrikson R.L., Vlodavsky I., Ilan N. (2003). Heterodimer formation is essential for heparanase enzymatic activity. Biochem. Biophys. Res. Commun..

[54] Fux L., Feibish N., Cohen-Kaplan V., Gingis-Velitski S., Feld S., Geffen C., Vlodavsky I., Ilan N. (2009). Structure-function approach identifies a COOH-terminal domain that mediates heparanase signaling. Cancer Res..

[55] Yang Y., Yuan F., Zhou H., Quan J., Liu C., Wang Y., Xiao F., Liu Q., Liu J., Zhang Y. (2023). Potential roles of heparanase in cancer therapy: Current trends and future direction. J. Cell. Physiol..

